# Practical application of VR in crisis-support training

**DOI:** 10.1192/j.eurpsy.2025.217

**Published:** 2025-08-26

**Authors:** H. Hooper

**Affiliations:** VirtualSpeech, Sunbury-on-Thames, United Kingdom

## Abstract

**Abstract:**

Psychological First Aid is (thankfully) not something most people use daily – yet it’s crucial for responders to be trained, confident and fully prepared for unexpected emergencies. So how can we ensure this readiness? The answer is AI avatars in virtual reality! At VirtualSpeech, in partnership with Region Västra Götaland, we’ve developed AI roleplay simulations that enable crisis and emergency teams to not only practice their psychological support skills but also receive personalized feedback on their performance and tips for improvement.

**Disclosure of Interest:**

None Declared

